# Whole-genome sequence of multi-drug resistant *Pseudomonas aeruginosa* strains UY1PSABAL and UY1PSABAL2 isolated from human broncho-alveolar lavage, Yaoundé, Cameroon

**DOI:** 10.1371/journal.pone.0238390

**Published:** 2020-09-04

**Authors:** Estelle Longla Madaha, Charlotte Mienie, Hortense Kamga Gonsu, Rhoda Nsen Bughe, Marie Christine Fonkoua, Wilfred Fon Mbacham, Kazeem Adekunle Alayande, Carlos Cornelius Bezuidenhout, Collins Njie Ateba

**Affiliations:** 1 Biotechnology Centre, Faculty of Science, University of Yaoundé 1, Yaoundé, Cameroon; 2 Laboratory of Bacteriology, Yaoundé University Teaching Hospital, Yaoundé, Cameroon; 3 Department of Disease, Epidemics and Pandemics Control, Ministry of Public Health, Yaoundé, Cameroon; 4 Bacteriology Service, Centre Pasteur du Cameroun, Yaoundé, Cameroon; 5 Unit for Environmental Sciences and Management, North-West University, Potchefstroom, South Africa; 6 Food Security and Safety Niche Area, Faculty of Natural and Agricultural Sciences, North-West University, Mmabatho, South Africa; Cornell University, UNITED STATES

## Abstract

*Pseudomonas aeruginosa* has been implicated in a wide range of post-operation wound and lung infections. A wide range of acquired resistance and virulence markers indicate surviving strategy of *P*. *aeruginosa*. Complete-genome analysis has been identified as efficient approach towards understanding the pathogenicity of this organism. This study was designed to sequence the entire genome of *P*. *aeruginosa* UY1PSABAL and UY1PSABAL2; determine drug-resistance profiles and virulence factors of the isolates; assess factors that contribute toward stability of the genomes; and thereafter determine evolutionary relationships between the strains and other isolates from similar sources. The genomes of the MDR *P*. *aeruginosa* UY1PSABAL and UY1PSABAL2 were sequenced on the Illumina Miseq platform. The raw sequenced reads were assessed for quality using FastQC v.0.11.5 and filtered for low quality reads and adapter regions using Trimmomatic v.0.36. The *de novo* genome assembly was made with SPAdes v.3.13 and annotated using Prokka v.2.1.1 annotation pipeline; Rapid Annotation using Subsytems Technology (RAST) server v.2.0; and PATRIC annotation tool v.3.6.2. Antimicrobial resistance genes and virulence determinants were searched through the functional annotation data generated from Prokka, RAST and PATRIC annotation pipelines; In addition to ResFinder and Comprehensive Antibiotic Resistance Database (CARD) which were employed to determine resistance genes. The PHAge Search Tool Enhanced Release (PHASTER) web server was used for the rapid identification and annotation of prophage sequences within bacterial genome. Predictive secondary metabolites were identified with AntiSMASH v.5.0. Clustered Regularly Interspaced Short Palindromic Repeats (CRISPR) and *cas* genes regions were also investigated with the CRISPRone and CRISPRFinder server. The genome sizes of 7.0 and 6.4 Mb were determined for UY1PSABAL and UY1PSABAL2 strains with G+C contents of 66.1% and 66.48% respectively. β-lactamines resistance genes *blaPAO*, aminoglycoside phosphorylating enzymes genes *aph(3’)-IIb*, fosfomycine resistance gene *fosA*, vancomycin *vanW* and tetracycline *tetA* were among identified resistance genes harboured in both isolates. UY1PSABAL bore additional *aph*(6)-Id, *aph(3'')-Ib*, ciprofloxacin-modifying enzyme *crpP* and ribosomal methylation enzyme *rmtB*. Both isolates were found harbouring virulence markers such as flagella and type IV pili; and also present various type III secretion systems such as *exoA*, *exoS*, *exoU*, *exoT*. Secondary metabolites such as pyochelin and pyoverdine with iron uptake activity were found within the genomes as well as quorum-sensing systems, and various fragments for prophages and insertion sequences. Only the UY1PSABAL2 contains CRISPR-Cas system. The phylogeny revealed a very close evolutionary relationship between UY1PSABAL and the similar strain isolated from Malaysia; the same trend was observed between UY1PSABAL2 and the strain from Chinese origin. Complete analyses of the entire genomes provide a wide range of information towards understanding pathogenicity of the pathogens in question.

## Introduction

*Pseudomonas aeruginosa* is an oxidase positive Gram-negative rod non-fermentative aerobic organism, mobile with a monotrichous polar flagellum [1]. It is an opportunistic pathogen isolated mostly in health care associated infections [2]. Like other Gram-negative bacteria, *P*. *aeruginosa* can express resistance through numerous mechanisms such as production of antibiotic modifying or degrading enzymes, active efflux pump and target modification [3]. Some strains show resistance to all clinically important antibiotics families. Strains resistant to at least three classes of anti-pseudomonas antibiotics are recognised as a multi-drug resistant *Pseudomonas aeruginosa* (MDR-PSA) [4]. The spread of MDR-PSA is increasing both within communities and hospital environments leading to severe infection cases and eventual therapeutic impasse [5]. Infections due to multidrug resistant pathogens impose overwhelming burden on the public health through increased hospital stay, cost of treatment, direct economic loss and death of patients from previously treatable disease conditions [6–9].

Persistence of MDR-PSA in the host environment is mainly due to various virulence factors expressed and reinforced by their multiple resistance mechanisms. Understanding the mechanisms involved in bacterial-host interaction can help to find new targets to thwart their spread. Shlaes [5] in his work: the perfect storm, mentioned that if the world strive to understand how resistance spread among microorganisms, then it could stop it and if the mechanisms by which the bacteria become resistant could be well scrutinized, we could find ways around them with discovery of novel antibiotics. Various tools have been developed to this effect and brought relevant knowledge concerning bacterial persistence and resistance.

Whole-genome sequencing (WGS) is one of the latest approaches to study resistance mechanisms in organisms. It gives massive information on the genes present within a pathogen, since this technique is capable of processing multiple DNA sequences in parallel with high throughput at reduced cost and time [10, 11]. It is a powerful protocol, which has revolutionized the study of microbial genomes. Next generation sequence has been used to investigate numerous genetic problems. To the best of our knowledge, there is no published data on *P*. *aeruginosa* whole-genome sequencing from clinical samples from Cameroon. Proper understanding of the evolution and physiology of these clinically relevant organisms will be archived by obtaining and comparing their complete genome. This study therefore evaluates the entire genomes of the two selected strains of MDR-PSA isolated from broncho-alveolar-lavage of human patients with lung infections in Yaoundé, Cameroon with a view to obtaining insight into the genes which are involved in the wide range resistance and virulence of the strains.

## Methodology

### Ethics statement

Ethical clearance was sought from the Central Region Ethical committee of the Central region, Cameroon, and an approval number (0259/CRESHC/2019) was assigned to the study. Administrative authorizations were obtained from the hospitals where bacteria isolates were obtained.

### Identification and selection of the bacteria strains

The bacterial strains were isolated from broncho-alveolar-lavage of human patients with lung infections and identified through PCR amplification of *Pseudomonas* species-specific 16S rRNA [F: GACGGGTGAGTAATGCCTA; R: CACTGGTGTTCCTTCCTATA] and *P*. *aeruginosa* specific 16S rRNA [F: GGGGGATCTTCGGACCTCA; R: TCCTTAGAGTGCCCACCCG] regions [2]. Both *P*. *aeruginosa* UY1PSABAL and *P*. *aeruginosa* UY1PSABAL2 were selected based on their multi-drug resistance profiling and ability to form strong biofilms on polystyrene at 25 and 37 °C from our previous study [12]. The susceptibility test was carried out using disk-diffusion method following the CASFM / EUCAST: Société Française de Microbiologie [13]. The test antibiotic drugs include: piperacillin (30μg); piperacillin-tazobactam (30/6μg); ticarcillin (75μg); ticarcillin-clavulanic acid (75/10μg); cefepime (30μg); ceftazidime (10μg); cefoperaxone (30μg); imipenem (10μg); meropenem (10μg); aztreonam (30μg); ciprofloxacin (5μg); levofloxacin (5μg); amikacin (30μg); gentamicin (10μg); netilmicin (10μg) and tobramycin (10μg).

### Complete genome sequence

The sequenced genomes of the MDR *P*. *aeruginosa* UY1PSABAL and UY1PSABAL2 were obtained through whole genome sequencing using an Illumina Miseq platform. One ng of the genomic DNA was tagmented with the Nextera XT DNA library prep kit according to the manufacturer’s protocol. The kit reagents fragment the DNA with simultaneous addition of adapter sequences. The libraries were amplified with a limited-cycle PCR program (12 cycles) to add the index 1 (i7) and index 2 (i5) adapters, containing sequences required for cluster generation of the Illumina flow cell. The library was purified using 0.6x Agencourt AMPure XP beads (Beckman Coulter). The quality and sizes of the resulting DNA fragments were evaluated on a 1.5% agarose gel. The libraries were quantified with a fluorometric method (Qubit, Life Technologies) and normalised to4 nM using a standard dilution method. The libraries were pooled, denatured with 0.1 N NaOH and diluted to the final loading concentration of 12 pmol. An identically treated PhiX control was added to a final concentration of 1%. Paired-end sequencing was done on an Illumina MiSeq platform using a MiSeq Reagent Kit V3 600 cycles.

### Quality control, trimming, assembling and annotation

The raw sequenced reads were assessed for quality using FastQC v.0.11.5 [14] and filtered for low quality reads and adapter regions using Trimmomatic v.0.36 [15]. The *de novo* genome assembly was made with SPAdes v.3.13 [16] and annotated using Prokka v.2.1.1 annotation pipeline [17]; Rapid Annotation using Subsytems Technology (RAST) server v.2.0 [18]; and PATRIC annotation tool v.3.6.2 [19].

### Identification of the resistome, virulome and Mobile Genetic Elements (MGEs)

Several clinically important antimicrobial resistance genes and virulence determinants were searched through the functional annotation data generated from Prokka, RAST and PATRIC annotation pipelines. In addition to ResFinder [20] and Comprehensive Antibiotic Resistance Database (CARD) [21] that were employed to determine the presence of resistance genes, the PHAge Search Tool Enhanced Release (PHASTER) web server [22] was used for the rapid identification and annotation of prophage sequences within bacterial genomes. The PHAge Search Tool Enhanced Release (PHASTER) web server [22] was used for the rapid identification and annotation of prophage sequences within bacterial genome. Predictive secondary metabolites were identified with AntiSMASH v.5.0 [23]. Clustered Regularly Interspaced Short Palindromic Repeats (CRISPR) and CRISPR-associated genes (*Cas*) were also investigated with the CRISPRone [24] and CRISPRFinder server [25].

#### Phylogeny

Evolutionary relationship between the strains UY1PSABAL and UY1PSABAL2; and twenty four other strains of randomly selected *P*. *aeruginosa* isolated from *Homo sapiens* between 2014 and 2016 across Africa, America, Asia and Europe was determined using PATRIC Fastree platform [19]. The Newick result file was then assessed using the Figtree v1.4.3 software to view the tree and annotation. The generated tree was labelled according to the codified name, which are in consistency with the strain’s country of collection and the GenBank accession number.

## Results

### Basic statistical features of the genome assembly

**Fig 1:**
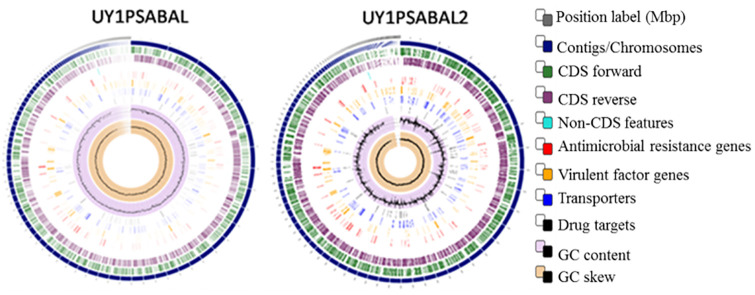
Circular genome mapping of the UY1PSABAL and UY1PSABAL2. The circular genome was generated with PATRIC sever v3.6.2.

The genome assembly size of the *P*. *aeruginosa* UY1PSABAL is 7,029,327 bp distributed into 136 contigs and having a G+C content of 66.1%. The number of protein-coding sequences within the genome is about 6,907, with 63 non-coding RNAs (including tRNAs and rRNAs). The N50 of the genome is 118,711 bp while the L50 is 18. On the other hand, the genome assembly size of the *P*. *aeruginosa* UY1PSABAL2 is 6,354,435 bp along 96 contigs with 66.48% G+C contents. The genome consists of about 6,046 protein-coding sequences and 63 non-coding RNAs. The genome’s N50 is 215,321 bp and the L50 is 10. The circular genome representing the entire genome assembly is shown in Fig 1.

### Antimicrobial resistance profiling

Phenotypic characterization of the antimicrobial resistance patterns of the *P*. *aeruginosa* UY1PSABAL and UY1PSABAL2 revealed that both strains have acquired resistance against a large proportion of clinically important antibiotics. The UY1PSABAL showed resistance against 60% of the test drugs while the UY1PSABAL2 was resistant against 41% but both strains displayed similar resistance patterns against 12% of the drugs tested (Table 1).

**Table 1:** Phenotypic antibiotic resistance patterns of UY1PSABAL and UY1PSABAL2 strains.

Antibiotics	Zones of inhibition		
	Recommendation [13]	UY1PSABAL	UY1PSABAL2
Piperacillin (30μg)	S≥18, R<18	12 (R)	6 (R)
Piperacillin-tazobactam (30/6μg)	S≥18, R<18	20 (S)	6 (R)
Ticarcillin (75μg)	S≥18, R<18	13 (R)	22 (S)
Ticarcillin-clavulanic acid (75/10μg)	S≥18, R<18	10 (R)	16 (R)
Cefepime (30μg)	S≥19, R<19	10 (R)	6 (R)
Ceftazidime (10μg)	S≥16, R<16	21 (S)	13 (R)
Cefoperaxone (30μg)	S≥21, R<14	19 (I)	6 (R)
Doripenem (10μg)	S≥25, R<22	30 (S)	27 (S)
Imipenem (10μg)	S≥20, R<17	23 (S)	21 (S)
Meropenem (10μg)	S≥24, R<18	30 (S)	22 (I)
Aztreonam (30μg)	S≥50, R<16	17 (I)	17 (I)
Ciprofloxacin (5μg)	S≥25, R<22	6 (R)	13 (R)
Levofloxacin (5μg)	S≥20, R<17	6 (R)	21 (S)
Amikacin (30μg)	S≥18, R<15	6 (R)	20 (S)
Gentamicin (10μg)	S≥15, R<15	6 (R)	16 (S)
Netilmycin (10μg)	S≥12, R<12	6 (R)	15 (S)
Tobramycin (10μg)	S≥16, R<16	6 (R)	17 (S)

Keys: R = Resistance; I = Indifference; S = Susceptible.

On the other hand, a number of resistance genes harboured within the genomes of the UY1PSABAL and the UY1PSABAL2 strains were identified through functional annotation generated from the ResFinder and CARD platforms. Nine predicted regions encoding for levofloxacin resistance genes, in addition to other coding sequences that confer resistance to quinolones such as *crpP*, *gyrA* (variant/mutant), *parE* (variant/mutant) was detected only within the genome of UY1PSABAL while *basS* was detected only in the UY1PSABAL2. Both strains harboured resistance genes to β-lactams, aminoglycosides, tetracyclines, fosfomycins and glycopeptides (Table 2).

**Table 2:** Different resistance genes found within UY1PSABAL and UY1PSABAL2 genomes.

Antibiotics	Putative resistance gens	
	*P*. *aeruginosa* UY1PSABAL	*P*. *aeruginosa* UY1PSABAL2
β-lactams	*BlaPAO*, *BlaOXA-*395	*BlaPAO*, *BlaOXA-*486, *BlaOXA*-50
Quinolone	*crpP*, *gyrA* (variant/mutant), *parE* (variant/mutant) and nine regions for levofloxacin resistance	None
Aminoglycoside	*aph(3’)-*IIb, *aph(3”)*-Ib, *aph(6’)-*Id, *rmtB*	*aph(3’)-*IIb
Tetracycline	*tet(G)*, Class B [*tet(A)_2*, *tetR*], Class C [*tet(A)_1*, *tet(A)_3*]	Class B [*tet(A)_2*], Class C [*tet(A)_1*]
Fosfomycin	*fosA*	*fosA*
Glycopeptide	Vacomycin type-B: *vanW*	Vacomycin type-B: *vanW*
Phenicol	*catB7*	*catB7*
Sulphonamide	*sulI*	*sulI*
Peptide antibiotic	None	*Bass*
Efflux pump	*emrE*, *cpxR*, *muxB*, *muxC*, *opmB*, *soxR*, *oprM*, *mexA*, *triB*, *mexG*	*emrE*, *cpxR*, *muxC*, *soxR*, *oprM*, *mexG*, *pmpM*, *opmH*, *mexC*, *mexJ*, *mexL*, *armR*, *mexA*, *oprN*, *mexF*, *triC*, *triA*, *bcr-1*, *mexH*, *mexI*, *opmD*

### Identification of virulent determinants

The protein-encoding sequences putative for important virulent factors such as flagella protein biosynthesis, adherence motility, endotoxin, Type IV pili Adherence Twitching motility, ion uptake, anti-phagocytosis (Serum resistance), and Type I, Type II, Type III and Type VI secretion systems among others were found common to both isolates (S1 Table).

### Identification of the Mobile Genetic Elements (MGE)

#### Transposable elements

A plethora of CDS putative for different transposable elements were found in both isolates (S2 Table). A total number of 29 coding sequences that are known to encode for transposable elements were found associated with *P*. *aeruginosa* UY1PSABAL, among which is a single phage transposase located between 20617–18833 nucleotide position along the reverse DNA strand. Transposase proteins InsN and InsO belonging to the same insertion sequence family IS911 were found common to both isolates at various positions. The insertion element IS407 coding for transposase proteins commonly associated with *Burkholderia multivorans* was only found within the genome of UY1PSABAL2.

#### Prophage and CRISPR arrays

Prophage search by PHASTER showed the presence of coding sequences (CDS) which are predictive for five intact, three questionable and two incomplete prophages in the UY1PSABAL. On the other hand, only one putative CDS was found each for intact, questionable and incomplete prophages within UY1PSABAL2. A search through functional annotation for prophage related genes revealed 263 phage related genes in UYPSABAL and 83 identified in UYPSABAL2. Analyses of both strains for CRISPR-Cas system revealed three CRISPR arrays with associate cas genes, only within UY1PSABAL2 (Table 3). The CRISPRone software, together with the CRISPR-Cas system, revealed six cas-protein encoding sequences: cas1, cas3, cas8f, cas5f, cas7f and cas6f within UY1PSABAL2 genome.

**Table 3:** CRISPR-Cas sequences found within UY1PSABAL2.

Contig no	CRISPR location	CRISPR length (bp)	No of repeats	Repeats length (bp)	Spacer counts
5	62269–62897	628	11	28	10
6	182441–183789	1348	23	28	22
6	192664–194308	1644	27	28	31

### Secondary metabolites

Determination of secondary metabolites within the two isolates revealed nine different secondary metabolites distributed on 17 gene clusters and 15 gene clusters within the genomes of UY1PSABAL and UY1PSABAL2 respectively. Many of the identified secondary metabolites are shared by both isolates. The secondary metabolites most frequently found in the isolates were peptide synthetase cluster (NRPS)–like betalactone cluster, phenazine, homoserine lactone, bacteriocin, non-ribosomal NRPS, NRPS–like, N-acetylglutaminylglutamine amide (NAGGN), thiopeptide and phenazine. The two NRPS clusters found in both isolates showed 100% similarity, to 2-amino-4-methoxy-*trans*-3-butenoic acid and pyochelin synthesis clusters. Cyclodipeptide synthetase (CDPS) is the secondary metabolite found only in UY1PSABAL2.

### Phylogeny

**Fig 2:**
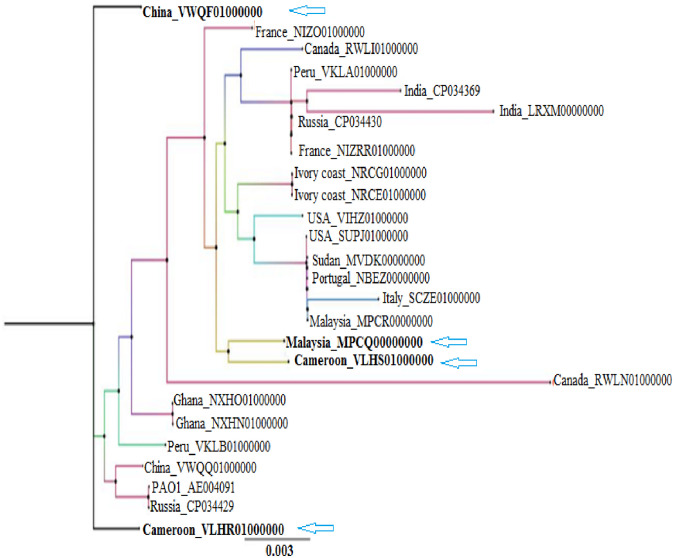
The phylogenetic tree showing the evolutionary similarity between UY1PSABAL and UY1PSABAL2 and other selected strains of *P*. *aeruginosa*. The tree was generated using PATRIC v3.6.2.

The phylogenetic tree revealing evolutionary relationship between UY1PSABAL and UY1PSABAL2 as compared with other selected strains of *P*. *aeruginosa* isolated from similar sources across continents is shown in Fig 2. A comparative analysis of the phylogeny showed that UY1PSABAL (Cameroon_VLHS00000000) and Malaysia_MPCQ00000000 belong to the same clade and both have about 0.9% divergence compared to the common ancestor. UY1PSABAL2 (Cameroon_VLHR00000000) strain and China_VWQQ01000000 belonged to the same monophyletic group and hence may have originated from a common ancestor. They are closely derived from the common ancestor with less divergence percentage (about 0.3%). Comparing with other strains sampled for this phylogenetic tree building, UY1PSABAL1 and UY1PSABAL2 strains are closely related to Asian strains.

## Discussion

Genome-based characterisation of isolates has provided solutions to several problems in species identification and pathogenicity. The entire genomes of the two MDR-PSA isolates used in this study were sequenced with a view to accessing their genetic make-up.

### Motility and bacteria adherence

Flagella are the most common apparatus used by bacteria for motility. Numerous encoding sequences putative for flagella formation were observed within the current isolates. This could server to improve their motility and consequently pathogenicity. Bacteria equipped with flagella are found to be more resistant to surfactant protein A (SP-A) [26] which is an important lung innate immune protein that kills microbial pathogens through opsonization and membrane permeability [27]. Both UY1PSABAL and UY1PSABAL2 were found equipped with flagella and this could have contributed to their resistance to opsonization by cells in the lung where the strains were isolated. In addition, specific pili such as type IV pili have been reportedly playing significant role in promoting motility and antimicrobial resistance in *P*. *aeruginosa* [27]. Piliated *P*. *aeruginosa* have been implicated in severe cases of pneumonia, bacteraemia, and increased mortality as compared to non-piliated strains [28]. Presence of this cellular component in both MDR-PSA strains under study could be associated with severity of lung infections [29].

### Cellular secretion systems

A number of secretion systems were identified common to both isolates. For instance, type II secretion systems that secrete folded proteins, such as pseudolysin (*lasB*); phospholipase C (*PlcH*); or lipase (*LipA*), from the periplasm into the extracellular milieu [30]. Type III secretion systems with robust virulence factor that allows translocation of specific bacterial effector proteins into the host cell cytosol or the plasma membrane [31, 32]. The presence of a functional T3SS was associated with bacterial persistence in the lungs, higher relapse rates, and increased mortality in patients with acute respiratory infections caused by *P*. *aeruginosa* [33]. Type VI (T6SS) is also an identified secretion systems that plays crucial role in competition and pathogenesis [34]. Both isolates contained T1SS, T2SS, T3SS and T6SS which are typical of *P*. *aeruginosa* secretion systems. These are channels by which the bacteria uptake or extrude molecules or toxins. Thus paved ways for the UY1PSABAL and UY1PSABAL2 to infect and persist in the host cells.

### Alginate production/regulation and anti-phagocytosis

Alginate serves as an extracellular matrix component involved in biofilm formation. It confers resistance to antibiotics and prevents phagocytosis by the immune cells such as macrophages of the host [35]. Synthesis of alginate is a complex process, involving twelve genes which are belong to the algA-algD operon [36]. Alginate production results in *P*. *aeruginosa* conversion from the non-mucoid to mucoid phenotype. Mucoid *P*. *aeruginosa* strains have been associated with a significant clinical deterioration. Beside protein-coding genes for alginate production, others anti-phagocytosis genes were equally found within the genomes of UY1PSABAL and UY1PSABAL2. These features represent important virulent traits in both isolates. There are novel therapeutic treatments targeting alginate synthesis [37], which can be a possibility for treatment in infections by UY1PSABAL and UY1PSABAL2.

### Quorum sensing

Bacteria can display different behaviours depending on cell density, and quorum sensing systems (QS) play a key role in sensing population density [38]. Homoserine lactone is the most studied quorum sensing auto-inducer molecule involved in the *las* and *rhl* QS systems. The *rhlI*, *lasI* homoserin lactone synthase, *pqsE Pseudomonas* quinolone system gene belonging to *rhl*, *las* and *pqs* QS system genes and *lasR* gene encoding for a transcriptional regulator were found in the UY1PSABAL and UY1PSABAL2 genomes. The QS plays a major role in virulence of *P*. *aeruginosa* [37]. It allows *P*. *aeruginosa*, for example, in the presence of high bacterial numbers to produce two *P*. *aeruginosa* toxins found within these isolates: an elastase (protease) and a pyocyanin (syderophore) [39], which will subsequently harm their host.

### Hydrolytic enzymes and ion uptake system

Proteases are enzymes with protein or peptide hydrolysis activity. Zinc metalloprotease such as *LasA* and *LasB* have elastolytic activity on human tissue, most especially in the lungs. Other hydrolytic enzymes, phospholipase C encoded by *plcH* and *plcN* genes can also be active on lung cells’ biomolecules such as Phospholipids contained in surfactants [40]. Among the phospholipases produced by *P*. *aeruginosa*, the thermo-stable haemolysin *PlcH* has the particularity of attenuating the reactive oxygen species (ROS) produced by neutrophils [31]. The genome of UY1PSABAL and UY1PSABAL2 contain the above-mentioned phospholipase C genes as well as zinc metalloprotease virulence factor genes. This further equipped the strains with capability to hydrolyse host cell proteins and avoid ROS produced by host immune cells, hence, could responsible for the bacterial persistence in the host environment.

Moreover, biological metal ions, including Co, Cu, Fe, Mg, Mn, Mo, Ni and Zn ions, are necessary for the survival and the growth of all microorganisms. They can serve as a cofactor in various biological mechanisms and are essential for microbial pathogenicity [41, 42]. Iron (Fe^3+^) is an essential element for living organisms. It is involved in numerous cellular processes of various organisms [43]. The two siderophores: pyochelin (*Pch*) and pyoverdine (*Pvd*) observed within UY1PSABAL and UY1PSABAL2 help in iron assimilation. Pyochelin (*Pch*) and pyoverdine (*Pvd*) chelate iron in the extracellular medium; the iron is then transported into the cell via a specific outer membrane transporter; *FptA* and *FpvA* respectively [42].

### Antibiotic resistance

The *P*. *aeruginosa* UY1PSABAL and *P*. *aeruginosa* UY1PSABAL2 were characterized as multidrug-resistant (MDR) after the phenotypic profiling revealed that both isolates are resistant to a wide range of clinically important antibiotics. This was further confirmed following the genomic analysis of the resistance genes (Table 2). Several published studies on *P*. *aeruginosa* genome characterization have highlighted the prevalence of *blaPAO* and *blaOXA50* which are classified as β-lactamases resistance genes [44, 45]; and this does not make a difference in the *P*. *aeruginosa* strains under study. In the study conducted by Girlich and colleagues [32], it was confirmed that insertion of *blaOXA-50* into a *P*. *aeruginosa* host, decreases susceptibility to ampicillin, ticarcillin, moxalactam and meropenem. Thus, presence of *blaOXA-50* within the UY1PSABAL2 genome could explain its resistance to meropenem. The genes *blaOXA-395* and *blaOXA-486* which are present respectively within UY1PASBAL and UY1PSABAL2, are commonly acquired resistance genes found within *P*. *aeruginosa* genome [45, 46].

Furthermore, quinolones resistance genes were also found common to both isolates. UY1PSABAL genome sequence showed nine predicted regions for levofloxacin and plasmid encoded ciprofloxacin resistance gene *crpP* which has been demonstrated to induce resistance against ciprofloxacin [47]. A *gyrA* variant/mutant and *parE* variant/mutant were observed within the same strain. The UY1PSABAL bearing a wide range of quinolone resistance markers explains its resistance to whole anti-pseudomonas quinolone (ciprofloxacin and levofloxacin) tested as compared to UY1PSABAL2, which did not carry any of these resistance genes or resistant variants, and eventually showed no resistance to quinolone antibiotics.

Additionally, *rmtB* is a 16S rRNA methylase which confers resistance to almost all aminoglycosides except streptomycin [48]. This ability was verified in UY1PSABAL and was confirmed harbouring the *rmtB* gene and consequently displayed resistance to all tested aminoglycosides (amikacin, gentamicin, gentamicin and tobramycin) represented by AMK^R^ GEN^R^ NET^R^ TOB^R^. Apart from the *rmtB* gene, aminoglycosides modifying enzymes (AMEs) were found within UY1PASBAL and UY1PSABAL2 genomes, and it was exclusively aminoglycoses phosphotransferases (APHs) that mostly confer resistance to amikacin and isepamicin [49]. The *aph(3’)-IIb* variant found in both UY1PASBAL and UY1PSABAL2 genome has been observed among MDR *P*. *aeruginosa* from several studies [44, 45, 50].

Identification of Class B and C tetracyclin; fosfomycin *fosA;* vancomycin B-type (*vanW*); and phenicols *catB7* resistance genes within the UY1PASBAL and UY1PSABAL2 genome suggest that these strains can express resistance to above-mentioned antibiotic families. The presence of vancomycin resistance gene within these genomes is worrisome because of the possibility of interspecies transfer [51] of this gene to *Staphylococcus aureus* for which vancomycin is the front line treatment antibiotic [52]. Efflux pump systems belong to five different classes. In agreement with several scientific reports in the literature [3, 53], Resistance-Nodulation-Division family (RND) was the most represented also within our isolate. And the efflux pump gene determinants were, as well, found in high numbers within both strains. It is important to note that efflux pump resistance determinants were found predominant within the UY1PSABAL2 strain.

### Mobile genetic elements and CRISPR-Cas system

Several coding sequences putative for mobile genetic elements are located within the genome of both isolates; these include, transposable elements and prophages. Transposable elements such as insertion sequences (IS) play a major role in the prokaryote genomes plasticity and are generally small mobile elements that typically carry one and sometimes two transposase (tnp) genes [54]. Transposase InsO, for IS911 and InsN transposase for IS407 (*Burkholderia multivorans*) were found within UY1PSABAL and UY1PSABAL2. Insertion sequence IS911, which is often located upstream of several β-lactamases [55], were also identified in both UY1PSABAL and UY1PSABAL2 strains. The IS407 has been demonstrated to promote the expression of silent *lac* operon in Transposons in *Burkholderia cepacia*. Transposons are DNA segments that can move from one genetic location to another. Transposon 4652 (Tn4652) identified within the genome of UY1PSABAL is a derivative of the toluene degradation transposon Tn4651 that belongs to the Tn3 which is a non-composite transposon bearing the β-lactamase resistance gene family of transposons [56]. The transposable elements found within the current isolates could have introduced the resistance genes that serve as the assets to resist antimicrobial agents, most especially β-lactams.

Clustered regularly interspaced short palindromic repeats (CRISPR) with associated cas genes is an adaptive immunity against mobile genetic elements, especially phages [57]. The combined results of PHASTER and RAST annotation showed a high number of intact prophage related genes in UY1PSABAL, indicating that various bacteriophage residues have infected this strain. The infection of UY1PSABAL was probably been made easier as a result of its lack of the “immune system” against bacteriophages and insertion sequences due to the absence of CRISPR-cas system. On the contrary, UY1PSABAL2 contained fewer prophages and IS related coding sequences; the presence of CRISPR-cas system might have protected the strain from invasion by bacteriophages.

### Secondary metabolites

Secondary metabolites are naturally produced compounds that serve as defensive molecules for the organisms producing them [58]. Various gene clusters coding for non-ribosomal peptide synthetases (NRPSs), non-proteinogenic peptides which are often toxic for prokaryotes and eukaryotes [59] were identified within the two isolates. Two NRPS clusters appeared to match with 100% similarity to clusters encoding 2-amino-4-methoxy-trans-3-butenoic acid and pyochelin within both UY1PSABAL and UY1PSABAL2 genomes. Some of the NRSPs were similar to pyoverdine cluster with less than 100% similarity. Pyoverdine is one of the common siderophores found within *P*. *aeruginosa* species and could represent a novel drug or vaccine target [37]. The *P*. *aeruginosa* toxin l,2-amino-4-methoxy-trans-3-butenoic acid (AMB) is a non-proteinogenic amino acid, which is toxic to prokaryotes and eukaryotes [60].

N-acetylglutaminyl-glutamine amide gene (NAGGN) cluster which plays a key role in cell osmoprotection of the bacterial cells were also observed. It is the most abundant solute at high salt concentration within *P*. *aeruginosa* [61]. β-lactone is another class of natural product found within these isolates. They have a unique reactivity among natural products and very suitable for the inhibition of hydrolases, transferases, ligases, and oxidoreductases unlike β-lactams which primarily target penicillin-binding proteins [62]. Phenazine is another secondary metabolite produced by *P*. *aeruginosa* and equally found within the genome of UY1PSABAL and UY1PSABAL2. Phenazine acts by altering the expression of immunomodulatory proteins by human airway epithelial cells [63].

Additionally, bacteriocin, the heat-stable ribosomally synthesized antibacterial peptides that are active by either killing or inhibiting the growth of other neighbouring bacteria [64] is also found within the genomes. This could serve as signalling peptide in the quorum sensing system [65]. Thiopeptide cluster is a biosynthetic gene cluster for a class of antibiotics widely distributed in genomes and metagenomes of the human microbial ecosystem [66]. Thiopeptides are known to be inactive against Gram-negative bacteria, nevertheless, thiostrepton; a thiopeptide antibiotic was found to stimulate *P*. *aeruginosa* biofilm formation [67]. This might have contributed to the production of biofilm exhibited by both isolates.

### Phylogeny

Analysis of the phylogenetic relationship between the UY1PSABAL and UY1PSABAL2 with other strains revealed that the strains under study were closely related to Asian (Malaysian and Chinese) strains. This could be attributed to the consequence of population dynamic. In fact, the last two decades were characterized with increased economic exchange between Asia and Africa; leading to increase in the cross-border travel between the two continents [68]. When populations move, they carry with them pathogens and cross-exchange of genetic material could occur. The phylogenetic proximity of our isolates to Malaysian and Chinese strains can be explained by the improved economic migration and social interaction between the two regions.

## Conclusion

This work focused on the characterisation of two MDR *P*. *aeruginosa* strains isolated from broncho-alveolar-lavage of lung-infected patients. The WGS approach was used to obtain a deeper insight into the complete DNA sequences of UY1PSABAL and UY1PSABAL2 strains. Numerous resistance and virulent genes observed in the genome of these isolates indicate their potential public implications, the severity of infections they could cause in humans and the difficulties in the management of these complications. The diverse MGEs identified in both strains demonstrate how dynamic each strain was during its evolutionary history. UY1PSABAL and UY1PSABAL2 strains are well equipped with secondary metabolites which serve as important asset for them to invade and persist in the host environment. Further studies are required to have a wider understanding of genetic features of *P*. *aeruginosa* isolates circulating within communities in Cameroon. This could ultimately bring about informed government policy and adequate community awareness toward curbing the transmission and continuous emergence of such dangerous pathogens.

## Supporting information

S1 Table Important virulence determinants located withinUY1PSABAL and UY1PSABAL2 genomes. (DOCX)

S2 Table Locations and sizes of identified transposable elements in *P*. *aeruginosa* UY1PSABAL and UY1PSABAL2. (DOCX)
