# Supplementary material for: Whole-genome sequence of multi-drug resistant Pseudomonas aeruginosa strains UY1PSABAL and UY1PSABAL2 isolated from human broncho-alveolar lavage, Yaoundé, Cameroon
Source: PLoS One. 2020 Sep 4;15(9):e0238390. doi: 10.1371/journal.pone.0238390 (PMC7473557; doi:10.1371/journal.pone.0238390)
Supplement: S1 Table — (DOCX) [file pone.0238390.s001.docx]

**S1 Table. Important virulence determinants located withinUY1PSABAL and UY1PSABAL2 genomes**

| **Identified genes for Virulent factors** | **Functions of the genes** | **Specific strain** |
| --- | --- | --- |
| *motA*, *motB*, *motD*, *flgB* | Flagellar motor protein biosynthesis, Flagella M-ring protein FilF | Both isolates |
| *flgI, fliF, fliR, fliE, fliQ, fliC, flgF, fliM, fliO, fleN, fliL, fliJ, flgG, fliG, flgK, fleR, flhF, flgE* | Adherence and motility | Both isolates |
| *flgP, fleH, fpvA* | Adherence and motility | UY1PSABAL |
| *fliD, fliH, flgJ, flgH, flgL, fleQ, fliN, flhA, flhB, flgD* | Adherence and motility | UY1PSABAL2 |
| *waaP, waaA, waaG, waaC, waaF* | Adherence and motility | Both isolates |
| *pilA, pilC, xcpA/pilD, pilF, pilG, pilH, pilJ, pilK, pilM, pilO, pilP, pilR, pilS, pilT, pilU, chpA, chpB, chpC, chpD* | Type IV pili Adherence Twitching motility | Both isolates |
| *pilX, pilE, pilI, pilB, pilW, fimU* | Type IV pili Adherence Twitching motility | UY1PSABAL |
| *chpE, fimV, pilI* | Type IV pili Adherence Twitching motility | UY1PSABAL2 |
| *aprF* | Protease exporter | Both isolates |
| *phzA* | Phenazin biosynthesis protein | Both isolates |
| *algR* | Alginate biosynthesis two-component system response regulator *algR* | Both isolates |
| *algK* | Alginate export system *algK*/*algE*, periplasmic component *algK* | Both isolates |
| *algQ* | Alginate regulatory protein *algQ* positive transcriptional regulator of *algD* | Both isolates |
| *phzCl* | 2-keto-3-deoxy-D-arabino-heptulosonate-7-phosphate synthase II *phzC* | Both isolates |
| *estA* | Phospholipase/lecithinase/hemolysin | Both isolates |
| *modA* | Molybdenum ABC transporter, substrate-binding protein *modA* | Both isolates |
| *lasR* | Homoserine lactone-binding transcriptional activator involved in quorum sensing | Both isolates |
| *narG* | (Respiratory nitrate reductase alpha chain) for biofilm formation | UY1PSABAL |
| *pchA, pchC, pchD, pchF, pchH, pchR, fptA, prdA, prdS* | Ion uptake | Both isolates |
| *pvdD, pvdE, fpvA* | Ion uptake | UY1PSABAL |
| *pchB, pchE, pchG, pchI* | Ion uptake | UY1PSABAL2 |
| *alg8, alg44, algA, algB, algE, algF, algG, algI, algJ, algK, algL, algR, algQ, algU, algX, algZ, mucB , mucC* | Antiphagocytosis (Serum resistance) | Both isolates |
| *mucA* | Antiphagocytosis (Serum resistance) | UY1PSABAL |
| *algP/algR3, algE* | Antiphagocytosis (Serum resistance) | UY1PSABAL2 |
| *exoT*, *toxA* | Toxin, Type III translocated protein, intracellular toxin, ADP-ribosyltransferase and GTPase activating | Both isolates |
| *exoU, exoY* | Toxin, Type III translocated protein, intracellular toxin, ADP-ribosyltransferase and GTPase activating | UY1PSABAL |
| *exoS* | Toxin, Type III translocated protein, intracellular toxin, ADP-ribosyltransferase and GTPase activating | UY1PSABAL2 |
| *aprF, lapC,* ATP-binding component *prtD/aprD, lapB, lapE* | Type I secretion system | Both isolates |
| *aprF* | Type I secretion system | UY1PSABAL |
| Outer membrane component *prtF*/*aprF* | Type I secretion system | UY1PSABAL2 |
| *xcpX, xcpV, xcpV, xcpW, xcpY, xcpS, xcpZ* | Type II secretion system | Both isolates |
| *xcpT* | Type II secretion system | UY1PSABAL |
| *xcpR, xcpP, xcpQ, xcpU* | Type II secretion system | UY1PSABAL2 |
| *exsA, exsB, exsC, exsD, pcr2, pcr3, pcr4, pcrH, pcrR, pcrV, popD, popN, pscB, pscC, pscD, pscE, pscF, pscG, pscJ, pscK, pscN, pscP, pscQ, pscU, pscS, pscR* | Type III secretion system | Both isolates |
| *lipI, pcrI, pscI, xcpX, pcrD* | Type III secretion system | UY1PSABAL |
| *exsE, popB, pscH, pscL, pscT, pscI, pscO* | Type III secretion system | UY1PSABAL2 |
| *tagR, vgrG1a, pppA, fha1, ppkA, hsiFI,vipB, hsiEI, hsiHI, icmF1/tssM1, lip1,hsiA1, hcp1, tagF/pppB, hsiJ1, clpV1, dotU1* | Type VI secretion system | Both isolates |
| *tagS, tagT, hisB1/vipA, PA0082* | Type VI secretion system | UY1PSABAL |
| *lasI, rhlI* | Regulation, quorum sensing system | Both isolates |
| *pqsE* | quinolone signal response protein, quorum sensing system | Both isolates |
| *LasA, lasB* | Extracellular zinc protease | Both isolates |
| *aprA* | Metalo-proteinase | Both isolates |
| *rh1A, rh1B* | rh1A rh1B TDP-rhamnosyltransferase | Both isolates |
| *phzH, phzM* | phenazine-specific methyltransferase phzH, phzM | Both isolates |
| *metE* | homocysteine methyltransferase | Both isolates |
| *phzG1* | Pyrixamine 5’-phosphate oxidase phzG | Both isolates |
| *phzD* | 2-amino-2-deoxy-isochorismate hydrolase phzD | Both isolates |
| *plcH* | Haemolytic plcH | Both isolates |
