# Supplementary material for: Whole-genome sequence of multi-drug resistant Pseudomonas aeruginosa strains UY1PSABAL and UY1PSABAL2 isolated from human broncho-alveolar lavage, Yaoundé, Cameroon
Source: PLoS One. 2020 Sep 4;15(9):e0238390. doi: 10.1371/journal.pone.0238390 (PMC7473557; doi:10.1371/journal.pone.0238390)
Supplement: S2 Table — (DOCX) [file pone.0238390.s002.docx]

**S2 Table.** Locations and the sizes of identified transposable elements in *P. aeruginosa* UY1PSABAL and *P. aeruginosa* UY1PSABAL2

| ***P. aeruginosa* UY1PSABAL** | | | ***P. aeruginosa* UY1PSABAL2** | | |
| --- | --- | --- | --- | --- | --- |
| **Transposable elements** | **Location** | **Size (bp)** | **Transposable elements** | **Location** | **Size (bp)** |
| Transposase InsN (IS911) | 1 - 63 | 63 | Transposase InsN (IS911) | 182212 - 182415 | 204 |
| Transposase InsN (IS911) | 2578 - 2637 | 60 | Transposase InsN (IS911) | 122464 - 122523 | 60 |
| Transposase InsN (IS911) | 8022 - 60 | 7963 | Transposase InsN (IS911) | 570256 - 570140 | 117 |
| Transposase InsO (IS911) | 782 - 666 | 117 | Transposase InsN (IS911) | 98554 - 98495 | 60 |
| Transposase InsO (IS911) | 53329 - 117 | 53445 | Transposase InsN (IS911) | 60 - 1 | 60 |
| Transposase InsO (IS911) | 3 - 1847 | 1845 | Transposase InsO (IS911) | 229 - 195 | 35 |
| Transposase InsO (IS911) | 15896 - 117 | 16012 | Transposase InsO (IS911) | 25428 - 25622 | 195 |
| Transposase InsO (IS911) | 860 - 828 | 33 | Transposase InsO (IS911) | 1118 - 1002 | 117 |
| Transposase insertion element IS407 | 198848 - 199111 | 264 | Transposase InsO (IS911) | 369 - 1 | 369 |
| Tn4652 transposase | 20371 - 3015 | 23385 | Transposase InsO (IS911) | 402 - 629 | 228 |
| Transposase and inactivated derivative | 11774 - 702 | 11073 | Transposase InsO (IS911) | 3 - 119 | 117 |
| Transposase and inactivated derivative | 170366 - 170968 | 603 | Transposase and inactivated derivative | 228 928 - 229362 | 435 |
| Transposase and inactivated derivative | 172316 - 172849 | 534 | Transposase and inactivated derivative | 28904 - 28302 | 603 |
| Phage transposase | 20617 - 18833 | 1785 | Transposase-like protein | 2730 - 3008 | 279 |
| TriA putative transposase | 2404 - 2805 | 402 | Transposase-like protein | 232153 - 232254 | 102 |
| Transposase | 65 - 3 | 63 | Transposase | 6148 - 6351 | 204 |
| Transposas | 1213 - 846 | 368 | Insertion element IS407 (Burkholderia multivorans) transposase | 190540 - 190277 | 264 |
| Transposase | 3 - 518 | 516 |  |  |  |
| Transposase | 55 - 819 | 765 |  |  |  |
| Transposase | 55615 - 55454 | 162 |  |  |  |
| Transposase | 55628 - 56437 | 810 |  |  |  |
| Transposase | 2 - 298 | 297 |  |  |  |
| Transposase | 365468 - 366343 | 876 |  |  |  |
| Transposase | 22259 - 22495 | 237 |  |  |  |
| Transposase | 5161 - 2967 | 2195 |  |  |  |
| Transposase | 64700 - 67585 | 2886 |  |  |  |
| Transposase | 15372 - 16307 | 936 |  |  |  |
| Transposase | 1405 - 2 | 1404 |  |  |  |
| Transposase | 5966 - 5175 | 792 |  |  |  |
